# HIF‐1α and HIF‐2α Are Upregulated in the Hippocampus but Not in the Medial Prefrontal Cortex in Experimental PTSD

**DOI:** 10.1002/brb3.71654

**Published:** 2026-08-05

**Authors:** Denys Porkhalo, Denis Pashevin, Yana Naumenko, Victor Dosenko

**Affiliations:** ^1^ Department of General and Molecular Pathophysiology Bogomoletz Institute of Physiology of NAS of Ukraine Kyiv Ukraine; ^2^ Department of Biophysics of Sensory Signalling Bogomoletz Institute of Physiology of NAS of Ukraine Kyiv Ukraine

**Keywords:** hippocampus, hypoxia‐inducible factors, pituitary adenylate cyclase‐activating peptide, PTSD, single prolonged stress

## Abstract

**Background:**

Post‐traumatic stress disorder (PTSD) is a psychiatric disorder characterized by anxiety, abnormal stress responses, and pathological memory formation. Research indicates that hypoxia‐inducible pathways might influence the neurobiology of PTSD. However, the specific relationship between hypoxia‐inducible factors (HIFs), neuropeptides, and brain regions remains unclear. This study investigated behavioral and molecular alterations in a rat model of PTSD, with a particular focus on the expression of HIF‐1α, HIF‐2α, HIF‐3α, PACAP, and PAI‐1 in the hippocampus and medial prefrontal cortex (mPFC).

**Methods:**

PTSD was modelled in adult male rats using the single prolonged stress (SPS) protocol, consisting of 2‐h immobilization, 15‐min forced swimming, and diethyl ether anesthesia. Behavioral assessment was performed 7 days post‐SPS using the open field test, elevated plus maze (EPM), and dark‐light box. Gene expression in the hippocampus and mPFC was quantified by RT‐qPCR using the 2^(−ΔΔCt)^ method with β‐actin as a reference gene.

**Results:**

SPS‐exposed rats exhibited significant anxiety‐like behavior. In the elevated plus maze, they showed increased freezing time (*p* = 0.0001), more freezing episodes (*p* = 0.002), fewer open‐arm head dips (*p* = 0.002), reduced open‐arm time (*p* = 0.027), and shorter total distance travelled (*p* = 0.026) compared to controls, whereas the reduction in open‐arm entries did not reach significance (*p* = 0.051). In the dark‐light box, PTSD animals made significantly fewer entries into the light zone (*p* = 0.009). No significant differences were detected in the open field test. At the molecular level, hippocampal HIF‐1α and HIF‐2α mRNA expression was significantly elevated in PTSD animals relative to controls (2.05‐ and 2.18‐fold, respectively; *p* < 0.05). No significant changes were detected for HIF‐3α, PACAP, or PAI‐1 in either brain region. No significant differences in gene expression were found in the mPFC.

**Conclusions:**

PTSD is associated with selective upregulation of HIF‐1α and HIF‐2α in the hippocampus, suggesting region‐specific activation of hypoxia‐inducible signaling pathways in the context of traumatic stress.

## Graphical abstract

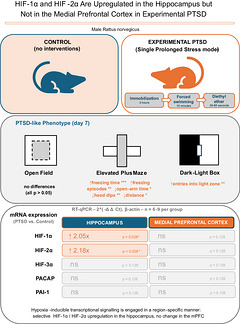

## 1 Introduction

It is well known that hypoxia‐inducible transcription factors (HIF‐1α, HIF‐2α) and HIF‐3α activate not only in response to reduced oxygen availability but also under other extreme conditions and coordinate adaptive responses, including metabolic reprogramming, angiogenesis, and neuroprotection. Unlike HIF‐1α and HIF‐2α, HIF‐3α is expressed as multiple splice variants: while some isoforms act as negative regulators of HIF‐1α and HIF‐2α, others retain transcriptional activity and can drive a distinct hypoxic gene program (Merelli et al. 2018; Sharp and Bernaudin 2004; Rius et al. 2008; Butt et al. 2021).

Are there HIFs potentially relevant components of the post‐traumatic stress disorder (PTSD) molecular landscape? Chronic psychological stress is accompanied by signs of oxidative stress and tissue hypoxia, mitochondrial dysfunction, which, in turn, can activate HIF‐dependent pathways (Chen et al. 2017). There is evidence that PACAP (Pituitary adenylate cyclase‐activating polypeptide) can modulate HIF expression, and HIFs can reciprocally influence PACAP receptor sensitivity (Singh et al. 2025; Stroth et al. 2011). At the neurobiological level, PTSD is associated with structural and functional changes in the hippocampus, amygdala, and medial prefrontal cortex (mPFC) (Bisson et al. 2015; Du et al. 2022). At the molecular level, the most noticeable change is a reduction in brain‐derived neurotrophic factor (BDNF) levels (a key regulator of neuroplasticity and memory consolidation) (Chen et al. 2017; Fujii et al. 2022). BDNF is not synthesized directly in its active form: its precursor, proBDNF, requires enzymatic activation via the tissue plasminogen activator (tPA) system. This system, in turn, is tightly regulated by the inhibitor PAI‐1, whose levels rise during stress and are associated with the suppression of neuroplasticity (Bouarab et al. 2021; Yang et al. 2024; Mennesson and Revest 2023). This raises the possibility of a feedback loop between hypoxic signaling and the PACAP–PAI‐1 axis, which could be of fundamental importance for understanding the molecular basis of PTSD. However, these relationships remain hypothetical for now.

This study aims to determine the expression levels of HIF‐1α, HIF‐2α, HIF‐3α, PACAP, and PAI‐1 in the hippocampus and mPFC from intact rats and rats with a PTSD‐like phenotype induced by the single prolonged stress (SPS) protocol (Muhie et al. 2017). A comparative analysis between the groups will determine whether these molecules undergo coordinated changes in response to SPS stress and assess the potential role of hypoxic signaling in the molecular pathogenesis of PTSD. The results obtained will form the basis for further investigation into the interaction among HIF, PACAP, and PAI‐1 as potential therapeutic targets.

## 2 Materials and Methods

### 2.1 Animals

Male rats, white outbred (*Rattus norvegicus*) (320–350 g), obtained from the animal facility (vivarium) of the Bogomoletz Institute of Physiology, National Academy of Sciences of Ukraine, were group‐housed with water and food available ad libitum. All experimental stages were performed according to the law of Ukraine “On the Protection of Animals from Cruelty” (Article 26; No 3447– IV) and European Convention for the Protection of Vertebrate Animals Used for Experimental and Other Scientific Purposes. All procedures were approved by the Committee for Biomedical Ethics of the Bogomoletz Institute of Physiology, NAS of Ukraine (protocol No. 1/26, dated February 11, 2026). All efforts were made to minimize animal suffering and reduce the number of animals used, in accordance with the 3Rs principles. The 12‐week‐old adult male rats were divided into two groups at random: a control group without any intrusions (*n* = 24) and a PTSD‐like group (*n* = 18).

### 2.2 Single Prolonged Stress

Prior to any experimental manipulation, the animals were maintained under standard vivarium conditions for a 7‐day acclimatization period, during which food and water were freely available, and a 12‐h light/dark cycle was kept.

**FIGURE 1:**
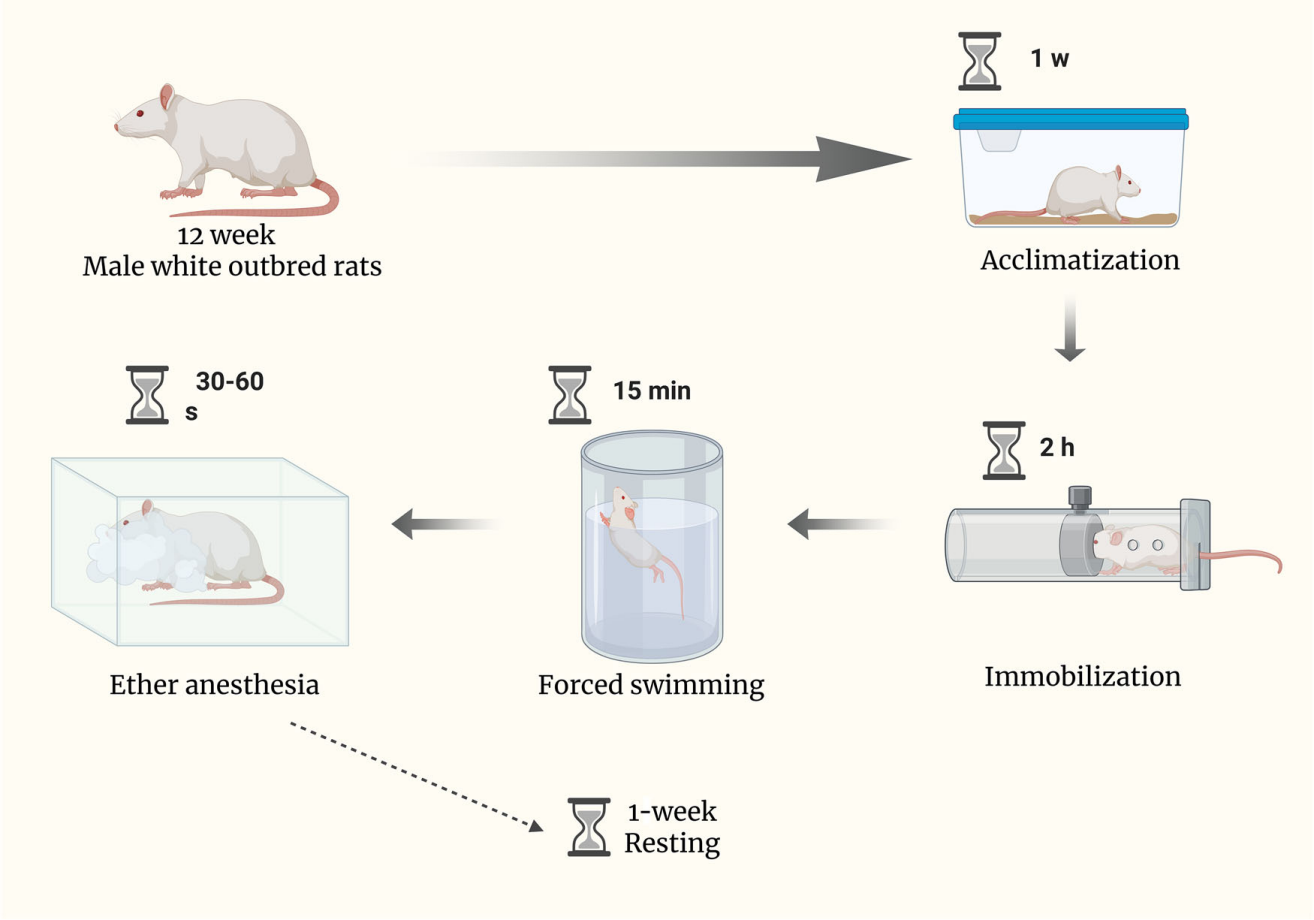
Experimental design. Male outbred white rats (12 weeks old) underwent 1 week of acclimatization, followed by the single prolonged stress (SPS) protocol, consisting of immobilization (2 h), forced swimming (15 min), and ether anesthesia (30–60 s). Animals were then left undisturbed for 1 week before behavioral and molecular assessments. Created in BioRender (https://BioRender.com/wk45p5r).

The SPS protocol was performed as a single‐day procedure comprising three sequential stressors (Lisieski et al. 2018), performed with three modifications relative to the original protocol (restraint, a 20‐min group swim, and ether exposure): immobilization was applied with the animal supine instead of by conventional restraint, the forced swim was carried out individually rather than in a group, and its length was shortened from 20 to 15 min. These changes allowed swimming behavior to be monitored on an individual basis and lessened the additional stress of group exposure. In practice, each animal was first immobilized for 120 min by laying it supine and fixing its limbs to a flat surface with adhesive tape, which blocked movement while permitting normal breathing. Immediately afterward, it underwent forced swimming for 15 min in an individual cylinder of room‐temperature water (22 ± 1°C). After a short drying interval, the animal was placed in a sealed chamber and exposed to diethyl ether vapor until the righting reflex was lost (roughly 30–60 s), under continuous observation to ensure unconsciousness without respiratory arrest. Once recovered from anesthesia, each animal was returned to its home cage and left undisturbed for 7 days (incubation period) before any further experimental procedures (Figure 1).

### 2.3 Behavioral Tests

Behavioral assessments were conducted 7 days following the SPS procedure, during the light phase of the light/dark cycle (09:00–15:00 h), in a dedicated testing room maintained at 22 ± 1°C under warm‐toned ambient lighting. Animals were transferred to the testing room 30 min before each session for acclimatization. Tests were administered in the following order: open field test (Seibenhener and Wooten 2015), elevated plus maze (EPM; Walf and Frye 2007), and dark‐light box (Bourin and Hascoët 2003), with a minimum of 24 h between successive tests to minimize carry‐over effects. The apparatus was thoroughly cleaned with 70% ethanol and allowed to dry between animals to eliminate olfactory cues. All sessions were video‐recorded and analyzed post hoc using the AnyMaze automated tracking software (Stoelting Co., USA).

#### 2.3.1 Open Field

General locomotion and anxiety‐related responses were evaluated in an opaque gray polyvinyl chloride open‐field arena measuring 75 × 75 × 40 cm. Each rat was released individually into the center of the arena and left to explore undisturbed for 10 min. For analysis, the floor was partitioned into a central zone, peripheral zones adjacent to the walls, and corner zones. The measures obtained comprised total path length (m), average movement speed (m/s), the number of entries into and time spent in each of these zones, the distance covered within the peripheral and corner zones, the count of freezing bouts, and the cumulative freezing duration(s); freezing was scored as a complete cessation of movement lasting at least 2 s.

#### 2.3.2 Elevated Plus Maze

The EPM comprised two open and two enclosed arms (each 50 × 11 cm) that met at a central 11 × 11 cm platform, with the whole apparatus spanning 110 × 110 cm and raised 60 cm above the floor. Each rat was positioned on the central platform, oriented toward an open arm, and permitted to explore freely for 5 min. The parameters captured were total distance travelled (m), the number of entries into and the time spent in the open and closed arms, the number of open‐arm head dips (scored when the animal extended its head beyond the edge of an open arm without advancing its body), the number of rotations, the number of freezing bouts, and the total freezing time(s). An arm entry was counted only when all four paws crossed into the respective arm.

#### 2.3.3 Dark‐Light Box

The box was formed by two compartments of equal size (each 56 × 56 × 40 cm), one illuminated and one dark, linked through a 7 × 7 cm aperture. The lit side was exposed to the ambient room light, whereas the dark side was screened from it. Each rat was introduced into the dark compartment, facing away from the aperture, and allowed to move freely for 5 min. The variables recorded were the number of crossings into and out of the lit compartment, the total time spent in the lit and in the dark compartments, and the number of curiosity behaviors, defined as the animal poking its head through the aperture into the lit side without a full‐body transition.

### 2.4 Tissue Collection

After anesthesia with ketamine (100 mg/kg, i.p.), the animals were euthanized by decapitation. The hippocampus and mPFC were dissected rapidly on ice, frozen in liquid nitrogen, and held at −80°C until processing. Because RNA extraction and RT‐qPCR (Reverse transcription‐quantitative polymerase chain reaction) profiling of five genes across two brain regions is a resource‐intensive endpoint, molecular analysis was performed on a representative subset of the behavioral cohort. Nine animals per group were selected at random, independently of behavioral performance (control, *n* = 24; PTSD, n = 18), and processed for gene‐expression analysis. The final molecular group sizes (*n* = 6–9) therefore reflect this planned subsampling, together with a small number of technical losses during tissue collection and RNA extraction (e.g., incomplete tissue recovery or extraction failure). Outlier removal (see Section 3) was applied as a separate step within these groups.

### 2.5 Molecular Analysis

#### 2.5.1 RNA Extraction

Total RNA was isolated with TRIzol reagent (Invitrogen, Thermo Fisher Scientific, USA) following the manufacturer's instructions. In brief, each frozen sample was homogenized in 1 mL of TRIzol, after which the phases were separated with chloroform, the RNA precipitated with isopropanol, and the pellet washed in 75% ethanol. The resulting pellets were dissolved in nuclease‐free water and their concentration determined spectrophotometrically. Sample purity was judged from the A260/A280 ratio, with values in the 1.8–2.0 range regarded as suitable for subsequent steps.

#### 2.5.2 CDNA Synthesis

First‐strand cDNA was generated from 1 µg of total RNA with the RevertAid First Strand cDNA Synthesis Kit (Thermo Fisher Scientific, USA), primed with random hexamers, as directed by the manufacturer. Reverse transcription was carried out without a preceding DNase step; instead, reaction specificity was verified by melt‐curve analysis of every primer pair, which produced a single peak in all samples. In addition, no‐reverse‐transcription (no‐RT) controls run for each primer pair gave no detectable signal, confirming that genomic DNA contamination was negligible.

#### 2.5.3 Primer Design and Synthesis

Gene‐specific primers for Hif1a, Epas1 (HIF‐2α), Hif3a, Adcyap1 (PACAP), and Serpine1 (PAI‐1) were designed for *R. norvegicus* target sequences. β‐Actin (Actb) was used as the reference gene for normalization. All primers were synthesized by Yuria‐Pharm (Ukraine) and validated for specificity by melt curve analysis before quantitative experiments. Amplicon sizes were verified using NCBI Primer‐BLAST against the *R. norvegicus* genome (taxid: 10116). Primer sequences, annealing temperatures, and amplicon sizes are provided in Table 1.

**TABLE 1:** Primer sequences used for RT‐qPCR analysis.

Gene	Protein	Forward primer (5'→3')	Reverse primer (5'→3')	Temperature (°C)	Amplicon (bp)
*Hif1a*	HIF‐1α	GCAACTGCCACCACTGATGA	GCTGTCCGACTGTGAGTACC	59.4/61.4	152
*Epas1*	HIF‐2α	ATGAGGCAGGACAGCAGGGG	GTCGGCCACCTGGAAGGTCT	63.5/63.5	111
*Hif3a*	HIF‐3α	CGTGGATGTATTCATAGGCAGA	GCCTGGACATGAAGTTCACATA	58.4/58.4	102
*Adcyap1*	PACAP	GCCTCTCTGGTTGTGATTCCA	GGTCATTCGCGGCTAGGAA	59.8/58.8	91
*Serpine1*	PAI‐1	CGTCTTCCTCCACAGCCATT	GTTGGATTGTGCCGAACCAC	59.4/59.4	97
*Actb*	β‐actin	TCATCACTATCGGCAATGAGC	GGCCAGGATAGAGCCACCA	57.9/61.0	302

*Note*: For each target gene, the table lists the gene symbol, corresponding protein, forward and reverse primer sequences (5'→3'), melting temperature (°C), and expected amplicon size (bp). β‐actin (Actb) was used as a reference gene for normalization.

#### 2.5.4 Quantitative PCR

Quantitative real‐time PCR was run on an Applied Biosystems 7500 Fast instrument with iTaq Universal SYBR Green Supermix (Bio‐Rad Laboratories). Each 10 µL reaction combined 5 µL of the SYBR Green supermix, 0.4 µL of each primer (10 pM), 1.5 µL of cDNA, and 2.7 µL of nuclease‐free water. Cycling began with a 10‐min denaturation at 95°C and continued through 40 cycles of 95°C for 15 s and 60°C for 1 min, ending with a melt‐curve step (60–95°C) to check amplicon specificity. Every sample was assayed in duplicate, and each plate carried a no‐template control (NTC). Expression was quantified by the 2^(−ΔΔCt)^ method, normalized to β‐actin, and reported as fold change relative to the Control group.

## 3 Statistical Analysis

All statistical analyses were performed using Python (version 3.12) with the SciPy (version 1.10) and NumPy (version 1.24) libraries. Before analysis, outliers were identified and removed using the interquartile range (IQR) method, with values falling outside the range (Q1 − 1.5 × IQR, Q3 + 1.5 × IQR) excluded from further analysis. This criterion corresponds to Tukey's fences, a standard, distribution‐free rule for outlier detection that does not assume normality and is therefore appropriate for the small group sizes and potentially skewed distributions in this study. The rule was defined a priori and applied uniformly and identically to every behavioral and molecular variable; no data point was removed selectively or on the basis of the hypothesis being tested. Outliers were evaluated separately within each group (control and PTSD) for each parameter, the number of excluded values was small (typically 0–2 per group per parameter), and all data points retained after outlier removal are displayed individually in the corresponding boxplots. The complete raw data set, together with the analysis code that implements this outlier procedure, is openly available (see the Data Availability statement), allowing full reproduction of every reported value.

Statistical analysis of behavioral data (open field test, EPM, dark‐light box) was performed separately for each test and each parameter. Before analysis, outliers were removed using the interquartile range (IQR 1.5×) method: values falling outside the range Q1 − 1.5 × IQR or Q3 + 1.5 × IQR were excluded from further analysis.

The normality of the distribution in each group was tested using the Shapiro–Wilk test (*α* = 0.05). For the two‐group comparison (control vs. PTSD), the two‐sample *t*‐test (Student's *t*‐test) was used when both groups met the normality criterion; when at least one group deviated from a normal distribution, the Mann–Whitney *U* test (two‐sided) was applied. The level of statistical significance was set at *p* < 0.05. The results are presented as mean ± SD; the graphs show the median, IQR, whiskers (1.5 × IQR) and individual animal values (boxplot with jitter).

Statistical analysis of relative gene expression data (HIF‐1α, HIF‐2α, HIF‐3α, PACAP, PAI‐1) was performed separately for each tissue (mPFC, hippocampus) and each gene. Outliers were removed within each group as described above for the behavioral data.

Normality of distribution was tested using the Shapiro–Wilk test (*α* = 0.05). For the two‐group comparison (control vs. PTSD), the independent samples *t*‐test was used when both groups were normally distributed; when either group deviated from normality, the Mann–Whitney *U* test (two‐sided) was applied. Significance level was set as follows: *p* < 0.05 (*), *p* < 0.01 (**), and *p* < 0.001 (***). Data are presented as mean ± SD; in the graphs, boxplots show individual values after outliers were removed.

## 4 Results

### 4.1 Behavioral Tests

#### 4.1.1 Elevated Plus Maze

**FIGURE 2:**
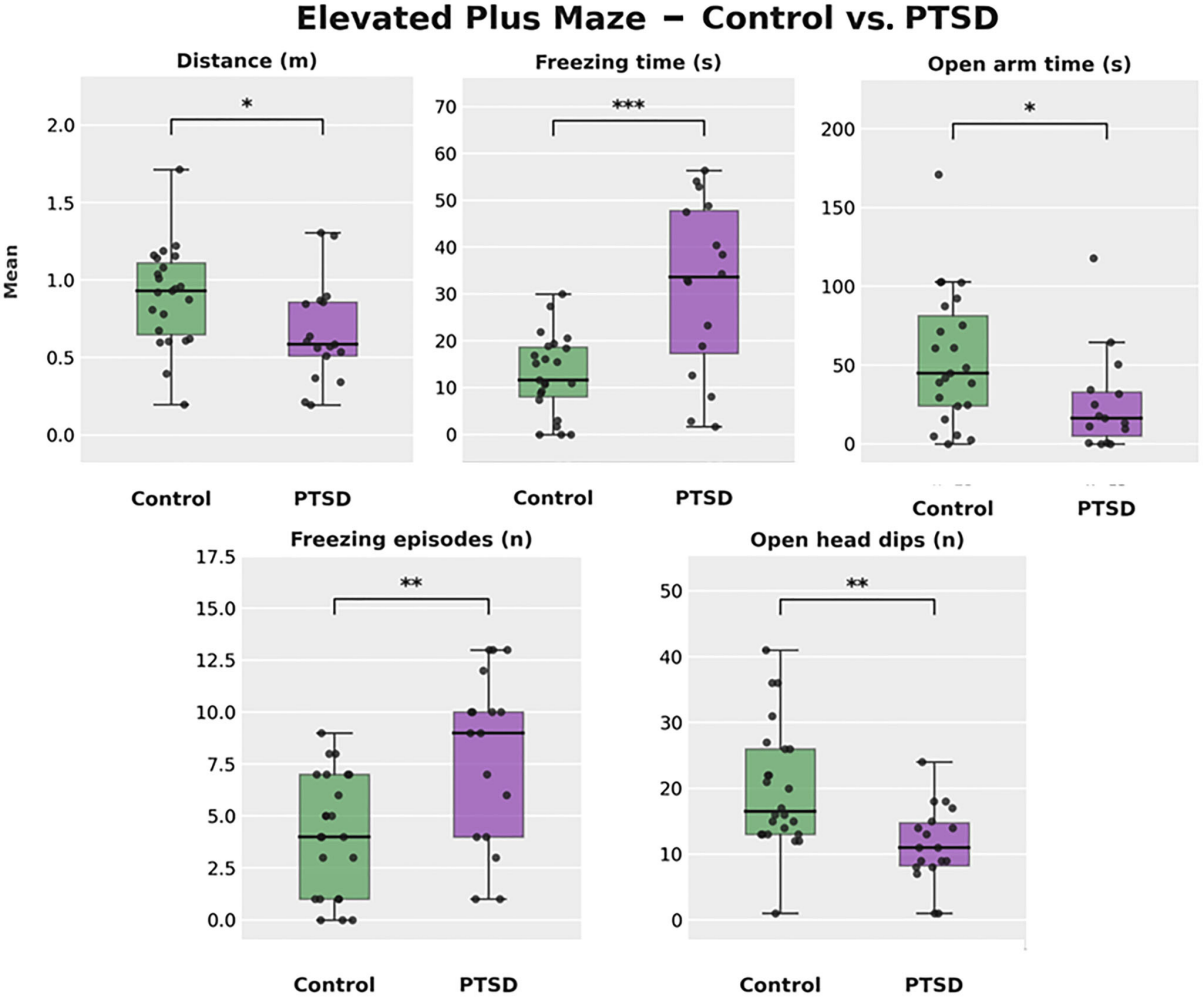
Elevated plus maze results—control versus PTSD. PTSD animals demonstrated significantly increased freezing time (*p* < 0.001) and number of freezing episodes (*p* < 0.01), reduced open arm time (*p* < 0.05), reduced open head dips (*p* < 0.01), and lower total distance travelled (*p* < 0.05) compared to controls. Boxplot: median, IQR, whiskers = 1.5 × IQR. Dots = individual animals (outliers removed, IQR 1.5×). *p < 0.05, **p < 0.01, ***p < 0.001.

Rats with PTSD exhibited significantly more anxiety‐like behavior compared with the control group. Freezing time in the PTSD group was approximately 2.5 times higher (31.61 s vs. 12.81 s in the control group; *p* = 0.0001), and the number of freezing episodes was almost twice as high (7.94 vs. 4.17; *p* = 0.002). Concurrently, a significant reduction in exploratory activity was observed: the number of open head dips decreased from 19.92 in the control group to 11.50 in the PTSD group (*p* = 0.002), open‐arm time was significantly reduced (26.19 s vs. 54.17 s; *p* = 0.027), and total distance travelled was significantly lower in the PTSD group (0.66 vs. 0.90 m; *p* = 0.026). The reductions in open‐arm entries (3.39 vs. 5.09; *p* = 0.051) and closed‐arm entries (7.44 vs. 10.78; *p* = 0.072) showed the same trend but did not reach statistical significance (Figure 2).

PTSD animals displayed a clear anxiety‐like behavioral profile in the EPM compared to controls. Specifically, PTSD rats showed significantly increased freezing behavior, as reflected by both longer freezing time and more freezing episodes, alongside a marked reduction in open arm exploration time. Total distance travelled was also significantly lower in the PTSD group. Together, these findings confirm successful induction of a PTSD‐like phenotype following the SPS protocol. Detailed statistics are provided in Table 2.

**TABLE 2:** Elevated plus maze results—control versus PTSD (mean ± SD).

Parameter	Control (mean ± SD)	PTSD (mean ± SD)	Test	*p* value	Significance
Distance (m)	0.897 ± 0.322 (*n* = 23)	0.658 ± 0.321 (*n* = 17)	*t*‐test	0.026	*
Freezing time (s)	12.809 ± 8.494 (*n* = 23)	31.613 ± 18.483 (*n* = 16)	*t*‐test	0.0001	***
Freezing episodes (*n*)	4.174 ± 2.902 (*n* = 23)	7.941 ± 4.100 (*n* = 17)	*t*‐test	0.002	**
Open arm entries (*n*)	5.087 ± 2.729 (*n* = 23)	3.389 ± 3.363 (*n* = 18)	MWU	0.051	ns
Open arm time (s)	54.174 ± 42.196 (*n* = 23)	26.193 ± 31.717 (*n* = 15)	MWU	0.027	*
Open head dips (*n*)	19.917 ± 9.362 (*n* = 24)	11.500 ± 5.854 (*n* = 18)	*t*‐test	0.002	**
Closed arm entries (*n*)	10.783 ± 5.300 (*n* = 23)	7.444 ± 6.242 (*n* = 18)	*t*‐test	0.072	ns
Closed arm time (s)	187.483 ± 59.147 (*n* = 23)	225.488 ± 56.618 (*n* = 16)	*t*‐test	0.052	ns
Rotations (*n*)	6.083 ± 1.586 (*n* = 24)	5.611 ± 2.146 (*n* = 18)	*t*‐test	0.417	ns

*Note*: Statistically significant differences were observed in distance travelled, freezing time, freezing episodes, open arm time, and open head dips. Comparisons: *t*‐test or Mann–Whitney U (MWU). *p < 0.05, **p < 0.01, ***p < 0.001.

Abbreviation: ns, not significant.

#### 4.1.2 Open Field Test

No statistically significant differences were observed between the control and PTSD groups in any of the parameters assessed in the open field test, including locomotor activity, freezing behavior, and time spent in distinct zones (all *p* > 0.05). Descriptive statistics and test results for all parameters are provided in Table 3.

**TABLE 3:** Open field test results—control versus PTSD (mean ± SD).

Parameter	Control (mean ± SD)	PTSD (mean ± SD)	Test	*p* value	Significance
Distance (m)	17.909 ± 3.471 (*n* = 21)	18.000 ± 5.054 (*n* = 16)	*t*‐test	0.948	ns
Speed (m/s)	0.028 ± 0.007 (*n* = 20)	0.028 ± 0.012 (*n* = 16)	MWU	0.610	ns
Freezing episodes (*n*)	11.524 ± 7.236 (*n* = 21)	15.750 ± 9.363 (*n* = 16)	*t*‐test	0.130	ns
Freezing time (s)	41.986 ± 34.089 (*n* = 21)	59.537 ± 41.998 (*n* = 16)	MWU	0.163	ns
Centre entries (*n*)	13.545 ± 7.645 (*n* = 22)	10.059 ± 8.219 (*n* = 17)	MWU	0.212	ns
Centre time (s)	24.659 ± 18.697 (*n* = 22)	17.259 ± 15.432 (*n* = 17)	MWU	0.165	ns
Corner entries (*n*)	37.000 ± 20.281 (*n* = 22)	31.765 ± 26.906 (*n* = 17)	MWU	0.387	ns
Corner time (s)	211.323 ± 168.908 (*n* = 22)	147.953 ± 133.886 (*n* = 17)	MWU	0.263	ns
Side entries (*n*)	43.273 ± 31.171 (*n* = 22)	36.529 ± 36.258 (*n* = 17)	MWU	0.610	ns
Side time (s)	136.023 ± 106.281 (*n* = 22)	113.353 ± 114.541 (*n* = 17)	MWU	0.436	ns

*Note*: No statistically significant differences were observed between the groups in any of the assessed parameters, including locomotor activity, freezing behavior, and zone‐specific metrics; comparisons: *t*‐test or Mann–Whitney U (MWU).

Abbreviation: ns, not significant.

#### 4.1.3 Dark‐Light Box

**FIGURE 3:**
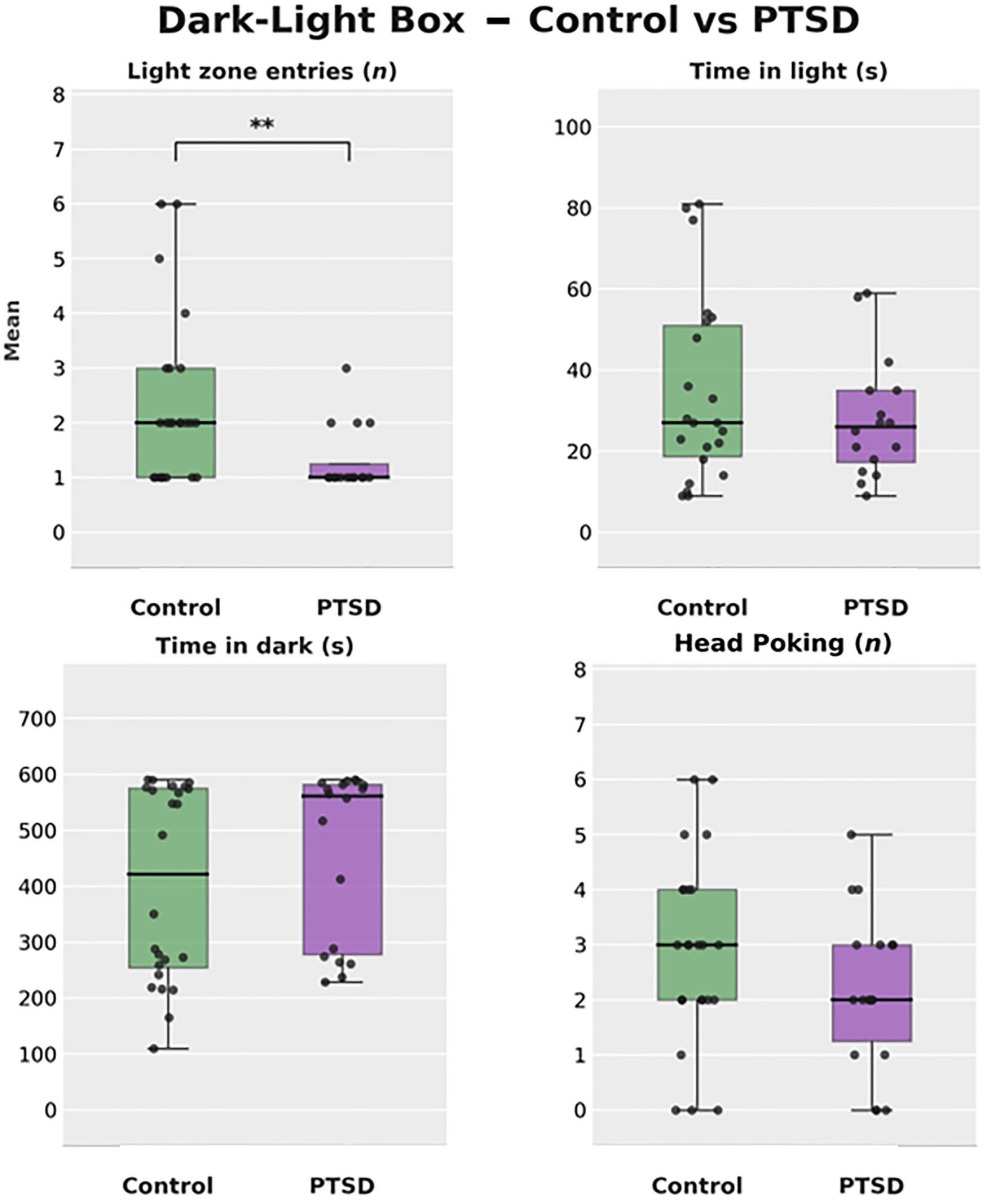
Dark‐light box results—control versus PTSD. PTSD animals showed significantly fewer entries into the light zone than controls (*p* < 0.01). No significant differences were observed in time spent in the light or dark compartments, or in curiosity behavior. Boxplot: median, IQR, whiskers = 1.5 × IQR. Dots = individual animals (outliers removed, IQR 1.5×). **p < 0.01.

In the dark‐light box, animals with PTSD exhibited diminished exploratory behavior relative to the control group, as demonstrated by a significantly lower number of entries into the light zone. No statistically significant differences were observed in the total time spent within the light or dark compartments. Additionally, head poking (curiosity‐related behavior) remained unchanged (Figure 3).

The only statistically significant difference between groups was observed in the number of light zone entries, which was markedly lower in PTSD animals than in controls (1.312 ± 0.602 vs. 2.333 ± 1.523; *p* = 0.009). Time spent in the light zone, time spent in the dark zone, and curiosity behavior did not differ significantly between the groups (all *p* > 0.05) (Table 4).

**TABLE 4:** Dark‐light box results—control versus PTSD (mean ± SD).

Parameter	Control (mean ± SD)	PTSD (mean ± SD)	Test	*p* value	Significance
Light zone entries (*n*)	2.333 ± 1.523 (*n* = 24)	1.312 ± 0.602 (*n* = 16)	MWU	0.009	**
Time in light (s)	34.500 ± 22.959 (*n* = 22)	27.938 ± 14.938 (*n* = 16)	MWU	0.604	ns
Time in dark (s)	403.625 ± 173.220 (*n* = 24)	459.611 ± 151.763 (*n* = 18)	MWU	0.227	ns
Head poking (*n*)	2.875 ± 1.702 (*n* = 24)	2.167 ± 1.425 (*n* = 18)	*t*‐test	0.161	ns

*Note*: A significant reduction in the number of light zone entries was observed in PTSD animals compared to controls (*p* = 0.009). No significant differences were found in time spent in the light or dark compartments, or in curiosity behavior. Comparisons: *t*‐test or Mann–Whitney *U* (MWU).

Abbreviation: ns, not significant.

**p < 0.01.

### 4.2 Molecular Analysis (qPCR)

#### 4.2.1 Hippocampus

A significant increase in HIF‐1α expression was observed in rats with PTSD: the mean value was 6.41 ± 3.79 relative units compared with 3.12 ± 1.43 in the control group (*p* = 0.028). A similar trend was observed for HIF‐2α: in the PTSD group, its level reached 57.72 ± 37.02, whereas in the control group, it was 26.42 ± 10.45 (*p* = 0.036). Thus, in the hippocampus, PTSD is associated with significant activation of both major isoforms of hypoxia‐inducible factors. No statistically significant changes in HIF‐3α were detected in a direct comparison between the control and PTSD groups (Table 5). The markedly higher absolute expression values of HIF‐2α compared with HIF‐1α likely reflect differences in primer amplification efficiency between assays; therefore, cross‐gene comparisons were based on fold‐change relative to the control group rather than on raw expression units.

**TABLE 5:** Hippocampal gene expression—control versus PTSD (mean ± SD).

Gene	Control (mean ± SD)	PTSD (mean ± SD)	*p* value	Significance	Test
HIF‐1α	3.1247 ± 1.4268 (*n* = 9)	6.4136 ± 3.7895 (*n* = 8)	0.028	*	*t*‐test
HIF‐2α	26.4160 ± 10.4509 (*n* = 8)	57.7165 ± 37.0170 (*n* = 9)	0.036	*	*t*‐test
HIF‐3α	1.1548 ± 1.2327 (*n* = 7)	4.4333 ± 4.9765 (*n* = 7)	0.128	ns	MWU
PACAP	3.6265 ± 1.6010 (*n* = 8)	4.6887 ± 3.2978 (*n* = 8)	0.426	ns	*t*‐test
PAI‐1	1.9061 ± 0.6485 (*n* = 6)	3.1821 ± 2.1391 (*n* = 6)	0.192	ns	*t*‐test

*Note*: Significantly elevated mRNA levels were detected for HIF‐1α (*p* = 0.028) and HIF‐2α (*p* = 0.036) in PTSD animals compared to controls. No significant differences were observed for HIF‐3α, PACAP, or PAI‐1. Comparisons: *t*‐test or Mann–Whitney *U* (MWU). *p < 0.05.

Abbreviation: ns, not significant.

**FIGURE 4:**
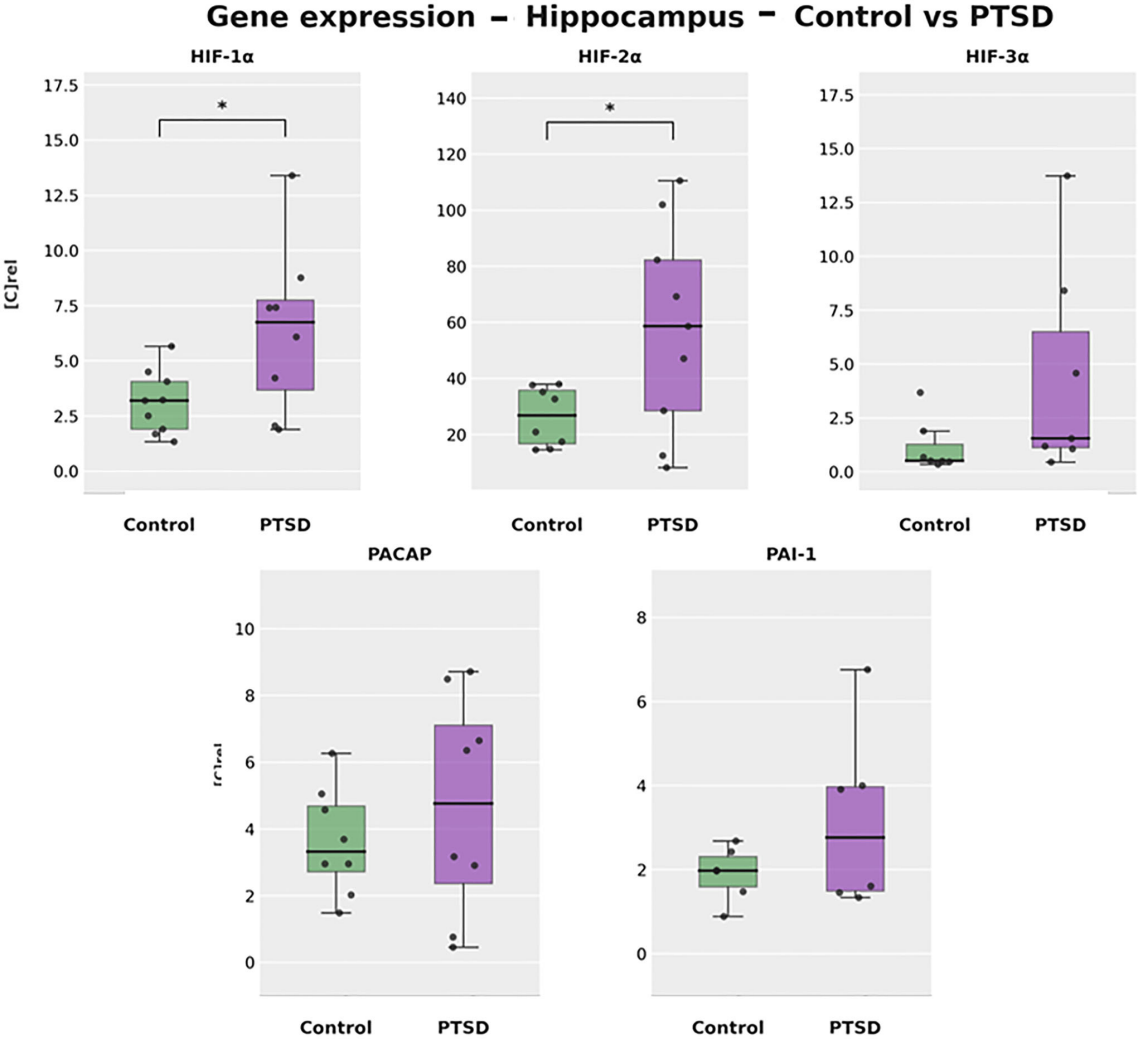
Hippocampal gene expression—control versus PTSD. PTSD animals exhibited significantly elevated mRNA levels of HIF‐1α (*p* < 0.05) and HIF‐2α (*p* < 0.05) compared to controls. No significant differences were found in HIF‐3α, PACAP, or PAI‐1 expression. Boxplot: median, IQR, whiskers = 1.5 × IQR. Dots = individual animals (outliers removed, IQR 1.5×). *p < 0.05.

PACAP and PAI‐1 levels in the hippocampus did not differ significantly between the control and PTSD groups (PACAP, *p* = 0.426; PAI‐1, *p* = 0.192). The distribution of individual PACAP values across groups is shown in Figure 4 (control vs. PTSD).

#### 4.2.2 Medial Prefrontal Cortex

In contrast to the hippocampus, no significant differences in gene expression were detected in the mPFC between the control and PTSD groups for any of the targets examined (all *p* > 0.05; Table 6).

**TABLE 6:** mPFC gene expression—control versus PTSD (mean ± SD).

Gene	Control (mean ± SD)	PTSD (mean ± SD)	*p* value	Significance	Test
HIF‐1α	3.014 ± 2.442 (*n* = 7)	4.512 ± 3.061 (*n* = 9)	0.211	ns	MWU
HIF‐2α	20.694 ± 7.183 (*n* = 6)	17.764 ± 6.248 (*n* = 7)	0.448	ns	*t*‐test
HIF‐3α	1.623 ± 1.602 (*n* = 5)	1.136 ± 0.816 (*n* = 5)	0.561	ns	*t*‐test
PACAP	3.871 ± 2.373 (*n* = 7)	3.289 ± 3.496 (*n* = 7)	0.722	ns	*t*‐test
PAI‐1	3.474 ± 3.309 (*n* = 6)	3.568 ± 2.412 (*n* = 7)	0.628	ns	MWU

*Note*: No statistically significant differences were observed between the groups in mRNA levels of HIF‐1α, HIF‐2α, HIF‐3α, PACAP, or PAI‐1 (all *p* > 0.05); comparisons: *t*‐test or Mann–Whitney U (MWU).

Abbreviation: ns, not significant.

## 5 Discussion

The behavioral results confirm the effectiveness of the SPS protocol in inducing a PTSD‐like state in rats. Importantly, the reduction in locomotion observed in the EPM (decreased total distance travelled) is unlikely to reflect a general motor deficit because total distance travelled and mean locomotion speed in the open field test were virtually identical between groups (17.9 vs. 18.0 m and 0.028 vs. 0.028 m/s, respectively; both *p* > 0.6). If SPS had impaired motor function, reduced locomotion would be expected across both tests. Instead, locomotion was selectively suppressed in the EPM—an apparatus that combines novelty with the aversive features of open, elevated arms—while remaining unaffected in the comparatively neutral open field. This context‐dependent pattern, together with the increased freezing and reduced open‐arm exploration in the EPM, indicates that the reduced movement reflects an anxiety‐driven, context‐specific behavioral inhibition rather than impaired locomotor capacity (Colucci et al. 2020).

In our study, we observed a significant increase in the expression of HIF‐1α and HIF‐2α in the hippocampus of rats with PTSD compared to controls. To our knowledge, this is among the first studies to report the upregulation of these hypoxia‐inducible factors in a rodent model of PTSD. This observation indicates that the SPS protocol is accompanied by the activation of transcriptional programs traditionally associated with tissue hypoxia and may be one of the key molecular pathways during PTSD pathogenesis. It should be noted, however, that the present study did not include a direct physiological readout of tissue hypoxia (e.g., pimonidazole staining or tissue oxygen measurements). The increased HIF‐1α and HIF‐2α mRNA levels are therefore interpreted as activation of hypoxia‐associated transcriptional programs rather than as direct evidence of a hypoxic state, and the relationship between HIF expression and actual tissue oxygenation in this model remains to be established.

HIF‐1α is responsible for the rapid adaptive response to a decrease in oxygen partial pressure, but HIF‐2α is primarily responsible for long‐term adaptation and vascular remodeling (Kumar and Choi 2015; Prabhakar et al. 2020). Therefore, HIF‐1α may reflect stress‐induced disturbances in neurovascular regulation and microcirculation in the hippocampus, and HIF‐2α indicates the chronic nature of these changes. These results are consistent with the concept that prolonged hyperactivation of the stress response leads to functional tissue hypoxia, mitochondrial dysfunction, neuronal hyperactivity, and so forth, in brain regions with high metabolic activity—primarily in the hippocampus (Conrad 2008; Doenyas‐Barak et al. 2023; Karanikas et al. 2021).

It is important to note that HIF‐1α and HIF‐2α have overlapping but not identical transcriptional targets. HIF‐2α predominantly regulates genes associated with stem cell properties, neurogenesis, and long‐term adaptation of the vascular bed (Martín‐Aragón Baudel et al. 2017). The simultaneous upregulation of both isoforms in PTSD may indicate the involvement of distinct but functionally related adaptive mechanisms.

The functional specificity of HIF‐3α may explain the absence of significant changes in this isoform in a direct comparison. HIF‐3α acts predominantly as a negative regulator of HIF‐1α and HIF‐2α, and its levels may only change in response to more pronounced or prolonged hypoxic stimulation than that induced by SPS stress. Although earlier studies reported that HIF‐3α mRNA expression increased rapidly after hypoxia, we did not observe a statistically significant change in HIF‐3α in the hippocampus (*p* = 0.128), which may reflect the milder and more transient hypoxic stimulus associated with SPS stress compared with the direct systemic hypoxia used in those studies (Heidbreder et al. 2003; Drevytska et al. 2012).

The absence of statistically significant differences in PACAP levels between the control and PTSD groups in the hippocampus may reflect the temporal specificity of PACAP regulation. PACAP levels may rise in the first hours and days following the stressor and return to baseline within 14 days after SPS (Granger et al. 2024; Ressler et al. 2011). It is possible that PACAP changes in the SPS model are more pronounced in the amygdala or entorhinal cortex than in the hippocampus, which is consistent with data on the regional specificity of PACAP‐dependent effects (Seiglie et al. 2022).

PAI‐1 showed no significant changes in either the hippocampus or the cortex. This may indicate that, at the transcriptional level, PAI‐1 remains stable in PTSD, while functionally significant changes occur predominantly at the protein or enzyme activity level. It is also possible that PAI‐1 responds to PTSD‐associated stimuli indirectly—via post‐translational regulation or a change in the tPA/PAI‐1 ratio, rather than through transcriptional mechanisms.

All significant molecular changes were limited to the hippocampus, with no changes observed in the mPFC. This regional specificity aligns with the hippocampus's known vulnerability to stress‐induced injury, due to its high metabolic demand, dense glucocorticoid receptor expression, and key role in HPA axis regulation and memory (Conrad 2008). The selective activation of HIF‐1α and HIF‐2α in specific regions confirms that the hippocampus is the primary site of stress‐induced molecular changes in the SPS model.

The present study provides a foundation for further investigation of hypoxia‐inducible and stress‐responsive molecular pathways in PTSD, and several directions warrant consideration in subsequent research. Collectively, these data establish a molecular basis for investigating HIF‐dependent signaling as a component of PTSD pathophysiology.

### 5.1 Limitations

This study has several limitations that should be considered when interpreting the results. First, our molecular analysis was restricted to the mRNA level. Because HIF proteins are predominantly regulated post‐translationally, mRNA changes may not fully reflect protein abundance or transcriptional activity; protein‐level validation by western blot or immunohistochemistry will be required to confirm these findings. Second, only a single reference gene (β‐actin) was used for normalization, whereas the MIQE (Minimum Information for Publication of Quantitative Real‐Time PCR Experiments) guidelines recommend the validation of at least two stable reference genes, particularly in stress studies where β‐actin itself may be affected; future studies should employ multiple reference genes to strengthen the reliability of fold‐change calculations. Third, the molecular analysis was performed on a relatively small number of animals per group (*n* = 6–9), which is smaller than the cohort used for behavioral testing. After outlier removal, group sizes fell as low as five to seven for some genes (notably HIF‐3α and PAI‐1); consequently, the nonsignificant molecular results may reflect insufficient statistical power rather than a true absence of effect, and these negative findings should be interpreted with caution. Fourth, only male rats were studied, so the generalizability of the findings to females remains uncertain. Fifth, gene expression was assessed at a single time point (7 days post‐SPS), which does not capture the temporal dynamics of HIF signaling. Sixth, the amygdala, a region centrally implicated in PTSD, was not analyzed. Finally, qPCR reactions were performed in duplicate; the MIQE guidelines recommend triplicate reactions for greater reliability. Cross‐gene comparisons of absolute expression values should also be interpreted with caution because of likely differences in primer amplification efficiency between assays. In addition, the study did not include a direct physiological marker of tissue hypoxia (e.g., pimonidazole staining or oxygen measurements), so the increased HIF expression reflects activation of hypoxia‐associated transcriptional programs rather than a directly measured hypoxic state. Finally, our behavioral characterization focused on the anxiety dimension of the PTSD phenotype; other PTSD‐related domains (such as fear conditioning, extinction, and hyperarousal) were not assessed and warrant investigation in future studies. These limitations should be addressed in future work to build upon the present findings.

## 6 Conclusions

In conclusion, exposure to single prolonged stress in male rats produced an anxiety‐like behavioral phenotype accompanied by a selective increase in HIF‐1α and HIF‐2α mRNA in the hippocampus, but not in the medial prefrontal cortex. These preliminary findings suggest that hypoxia‐associated transcriptional signaling may be engaged in the hippocampus under conditions of traumatic stress. However, given the exploratory nature of the study, the mRNA‐level analysis, and the limited sample size, these results should be interpreted with caution and require confirmation at the protein level before any mechanistic role in PTSD can be established.

## Author Contributions

**Denys Porkhalo**: Writing – original draft, Writing – review and editing, visualization, methodology, data curation, resources, formal analysis. **Victor Dosenko**: supervision, conceptualization, writing – review and editing, project administration. **Denis Pashevin**: conceptualization, writing – review and editing, validation. **Yana Naumenko**: writing – review and editing, visualization, validation.

## Funding

The authors have nothing to report.

## Ethics Statement

All experimental stages were performed according to the law of Ukraine “On the Protection of Animals from Cruelty” (Article 26; No. 3447 – IV) and European Convention for the Protection of Vertebrate Animals Used for Experimental and Other Scientific Purposes (Strasbourg, 1986). Ethical compliance of the research was approved with the Institutional Ethics Committee of the Bogomoletz Institute of Physiology, NAS, Ukraine (protocol No. 1/26, dated February 11, 2026).

## Conflicts of Interest

The authors declare no conflicts of interest.

## Data Availability

The data used for this study are available at Zenodo using the link https://zenodo.org/records/19283737 (https://doi.org/10.5281/zenodo.19283737). The repository additionally includes the per‐sample Ct values for every gene and animal, together with a script that recomputes all reported expression values from these raw data by normalizing each target gene to the β‐actin reference gene (the standard ΔCt/relative‐expression method), so that every value in the manuscript can be reproduced directly from the measurements. Code used for statistical analysis is available at https://github.com/DotardOneClick/PTSD_hypoxia_markers.
